# Bicarbonate supplementation enhances growth and biochemical composition of *Dunaliella salina* V-101 by reducing oxidative stress induced during macronutrient deficit conditions

**DOI:** 10.1038/s41598-018-25417-5

**Published:** 2018-05-03

**Authors:** Ramachandran Srinivasan, Anbazhagan Mageswari, Parthiban Subramanian, Chandrasekaran Suganthi, Amballa Chaitanyakumar, Velmurugan Aswini, Kodiveri Muthukalianan Gothandam

**Affiliations:** 1 School of Bio-Sciences and Technology, Vellore Institute of Technology, Vellore, 632 014 Tamil Nadu India; 2 Department of Agricultural Biotechnology (Metabolic Engineering Division), National Institute of Agricultural Sciences, Rural Development Administration, Jeonju, Republic of Korea

## Abstract

The unicellular marine alga *Dunaliella salina* is a most interesting green cell factory for the production of carotenes and lipids under extreme environment conditions. However, the culture conditions and their productivity are the major challenges faced by researchers which still need to be addressed. In this study, we investigated the effect of bicarbonate amendment on biomass, photosynthetic activity, biochemical constituents, nutrient uptake and antioxidant response of *D. salina* during macronutrient deficit conditions (N^−^, P^−^ and S^−^). Under nutrient deficit conditions, addition of sodium bicarbonate (100 mM) significantly increased the biomass, carotenoids including β-carotene and lutein, lipid, and fatty acid content with concurrent enhancement of the activities of nutrient assimilatory and carbonic anhydrase enzymes. Maximum accumulation of carotenoid especially β-carotene (192.8 ± 2.11 µg/100 mg) and lipids (53.9%) was observed on addition of bicarbonate during nitrate deficiency compared to phosphate and sulphate deficiency. Supplementation of bicarbonate reduced the oxidative stress caused by ROS, lowered lipid peroxidation damage and improved the activities of antioxidant enzymes (SOD, CAT and APX) in *D. salina* cultures under nutrient stress.

## Introduction

*Dunaliella* an edible marine microalgae is one of the largest sources of β-carotene (14% of dry weight of cell). It is a well-organized cell factory, it is mainly cultivated for its valuable biomolecules such as carotenoids, lipids and fatty acids^1^. Accumulation of useful molecules in *D. salina* can be improved by exposing the microalgal cultures to stress by altering several culture conditions including nutrient concentrations (excess or deficit), osmotic pressure fluctuations, temperature fluctuations, light intensity, radiation and pH fluctuations. Despite these stress conditions initially improving the amount of desired product, they are ultimately detrimental as they limit the overall growth, biomass and the productivity of the algal system due to slowing down the photosynthetic carbon fixation, inhibition of protein synthesis, induction of ROS (reactive oxygen species) in the cell that causes structural defects in chlorophyll, light-harvesting complex and RuBisCO (Ribulose-1,5-bisphosphate carboxylase/oxygenase). This eventually fails to balance the cost of production at an industrial scale^2^.

Among these stresses, nutrient-deficiency imposed by decreasing concentration of either individual components or combination of multiple components has been found to be economically profitable, and remains to be a potential area of research to improve industrial production of *D. salina* and its products^3–5^. Imposing nutrient-stress can transiently improve biomolecule accumulation, but in it long term, affects the overall production by hindering active cell growth. To overcome this problem, several studies have been carried out on the supplementation of inorganic carbon in the form of gas or solids. Carbon dioxide can be taken up and utilized by microalgae in two forms, namely inorganic carbon (sodium bicarbonate) and carbon-dioxide gas. Only a few algae have the ability to directly take up gaseous CO_2_ (carbon dioxide) for its growth, while others convert gaseous carbon into bicarbonate through chemical disequilibrium and utilize it for photosynthesis^6^. Solid substrate such as bicarbonate has been found to be more effective in marine microalgae than in freshwater microalgae under nutrient depleted conditions^6,7^. Sodium bicarbonate has also been found to increases their biochemical composition in nutrient deplete cultures thus making sodium bicarbonate, a convenient to transport and cost effective component providing a feasible alternative to gaseous inorganic carbon^7^. Only a limited number of microalgae have been investigated on the effect of sodium bicarbonate addition along with nitrate and phosphate deficient cultures and no report exists studying the effect of sodium bicarbonate under sulphate deficit growth conditions^7–9^.

Under normal conditions, the reactive oxygen species (ROS) rate is lowered in photosynthetic cells whereas in nutrient depletion, microalgae can produce high levels of ROS (H_2_O_2_, O_2_^−^ or OH^−^) which cause damage to cellular environment. ROS is known to be an important factor in cellular response and its detrimental role at higher concentrations has been well-established in photosynthetic microalgae under nutrient stress^10^. To mitigate and repair damage initiated by ROS during abiotic stress, algae have developed a defense mechanism, using a complex antioxidant systems^11–14^. However, in a few microalgal species, it has been reported that the ROS scavenging antioxidant enzyme in microalgae is influenced by bicarbonate^15,16^. To our knowledge, this is the first report studying the effect of bicarbonate amendment on antioxidant response of *D. salina* during nutrient deficit conditions.

Our study focuses on evaluating the combined effect of sodium bicarbonate and macronutrient starvation such as nitrate (N^−^), phosphate (P^−^) and sulphate (S^−^) stress on physiological and biochemical response of *D. salina*.

## Results

### Growth, pH, biomass and photosynthetic activity

**Figure 1:**
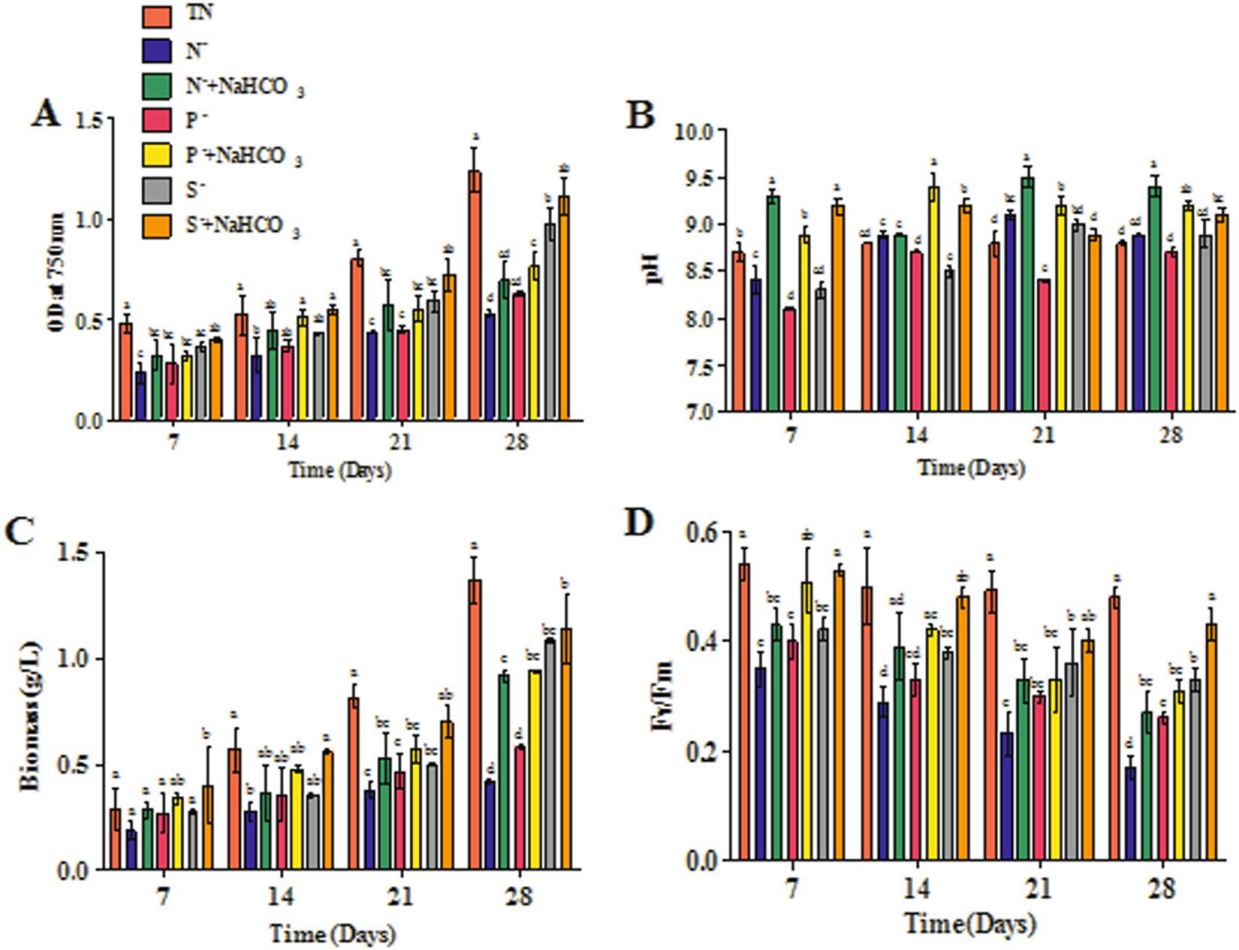
Effect of bicarbonate on growth (**A**), pH (**B**), biomass (**C**) and photosynthetic activity (**D**) of *D. salina* under nutrient deficit conditions. All values are expressed as Mean ± SD (n = 3). Values with different letters represent significantly differ at p < 0.05 between the groups. Total Nutrient (TN); Nitrate deficit (N^−^); Nitrate deficit with bicarbonate (N^−^ + NaHCO_3_); Phosphate deficit (P^−^); Phosphate deficit with bicarbonate (P^−^ + NaHCO_3_); Sulphate deficit (S^−^); Sulphate deficit with bicarbonate (S^−^ + NaHCO_3_).

Physiological parameters of *D. salina* during macronutrient stressors such as N^−^, P^−^ and S^−^ with or without amendment of bicarbonate were studied for 28 days (Fig. 1). The *Dunaliella* cells were subjected to these three different nutrient stresses in the presence and absence of sodium bicarbonate (100 mM). Figure 1A indicates the effect of sodium bicarbonate on the growth of *D. salina* under nutrient deficit conditions (OD_750_). These results showed that the growth of *Dunaliella* was significantly affected in the macronutrient deficit cultures. However, the media amended with 100 mM sodium bicarbonate showed significant recovery in the cell growth by reducing the oxidative stress effects during nutrient deficit conditions. The maximum cell growth recovery was observed in the sulphate deficit medium amended with sodium bicarbonate (S^−^ + NaHCO_3_ – 1.11 ± 0.09) followed by phosphate deficit medium with sodium bicarbonate (P^−^ + NaHCO_3_ – 0.70 ± 0.07) and nitrate deficit medium with sodium bicarbonate (N^−^ + NaHCO_3_ – 0.65 ± 0.09) showed the least recovery.

The pH of all the macronutrient deficit cultures with sodium bicarbonate varied from 9.1 to 9.4 at the end point of experiment (Fig. 1B). pH of nutrient deficit *Dunaliella* cultures with bicarbonate (N^−^ + NaHCO_3_–pH 9.3 ± 0.07, P^−^ + NaHCO_3_–pH 8.9 ± 0.09, S^−^ + NaHCO_3_–pH 9.2 ± 0.08) was significantly higher than those of with no bicarbonate (N^−^–pH 8.4 ± 0.15, P^−^–pH 8.1 ± 0.01, S^−^–pH 8.3 ± 0.08), after 7 days of incubation.

Figure 1C represents the effect of bicarbonate amendment on biomass of *Dunaliella* cultures under nutrient deficit condition. Removal of macronutrient from the growth medium significantly affected the metabolic process of *Dunaliella* and resulted in lower biomass (N^−^–0.42 ± 0.01 g/l and P^–^–0.58 ± 0.01 g/l), whereas biomass production of *D. salina* remained largely unaffected in S^−^ (1.08 ± 0.01 g/l) and S^−^ + NaHCO_3_ (1.14 ± 0.16 g/l) as compared to the total nutrient cultures (1.37 ± 0.11 g/l). However, addition of bicarbonate on the macronutrient deficit medium (N^−^ + NaHCO_3_–0.92 ± 0.03 g/l, P^−^ + NaHCO_3_–0.94 ± 0.01 g/l, S^−^ + NaHCO_3_–1.14 ± 0.16 g/l) significantly enhanced the biomass of *D. salina*, which indicates the active utilization of bicarbonate for the growth and metabolic process of microalgae under stress conditions. The effect of bicarbonate on photosynthesis efficiency (F*v*/F_m_) of *Dunaliella* cultures under nutrient deficit conditions were presented in Fig. 1D. Maximum photosynthesis efficiency was observed in the nutrient deficit *Dunaliella* cultures amended with bicarbonate, whereas all nutrient deficit cultures (N^−^, P^−^ and S^−^) showed decreased photosynthetic efficiency compared to control containing total nutrients. The addition of bicarbonate showed significant increase in the photosynthetic efficiency (F*v*/F_m_) of *Dunaliella* cultures during nutrient deficit conditions.

### Photosynthetic pigments

In the present study, we measured the photosynthetic pigments chlorophyll-a, chlorophyll-b and total carotenoid content in all the experimental samples (Table 1). The observations showed that the ratio of chlorophyll a/b in nutrient deficit cultures was much lower compared to depleted culture amended with sodium bicarbonate. As shown in Table 1, addition of sodium bicarbonate to the nitrate and phosphate deficit cultures (N^−^ + NaHCO_3_–1.02 ± 0.12 µg/mL, P^−^ + NaHCO_3_ – 0.98 ± 0.05 µg/mL) showed significant increase in photosynthetic pigments compared to nutrient deficit cultures (N^−^–0.52 ± 0.01 µg/mL and P^−^–0.55 ± 0.03 µg/mL). Pigment content of S^−^ cultures was less affected (1.32 ± 0.03 µg/mL and 1.41 ± 0.15 µg/mL chlorophyll-a) irrespective of the presence of sodium bicarbonate.

**Table 1:** Effect of bicarbonate on photosynthetic pigments of *D. salina* under nutrient deficit conditions.

Treatments	Chl-a (µg/mL)	Chl-b (µg/mL)	Chl a + b (µg/mL)	Car (µg/mL)	Chl a/b	Car/Chl a + b
TN	1.56 ± 0.05^a^	1.29 ± 0.06^a^	2.85 ± 0.01^a^	4.62 ± 0.02^f^	1.21 ± 0.08^d^	1.62 ± 0.11^f^
N^−^	0.52 ± 0.01^d^	0.21 ± 0.01^e^	0.73 ± 0.03^e^	7.23 ± 0.08^b^	2.48 ± 0.01^a^	9.9 ± 0.02^a^
N^−^ + NaHCO_3_	1.02 ± 0.12^bc^	0.47 ± 0.04^d^	1.49 ± 0.09^d^	8.25 ± 0.01^a^	2.17 ± 0.06^b^	5.54 ± 0.09^c^
P^−^	0.55 ± 0.03^d^	0.22 ± 0.02^e^	0.77 ± 0.02^e^	5.43 ± 0.01^d^	2.50 ± 0.01^a^	7.05 ± 0.01^b^
P^−^ + NaHCO_3_	0.98 ± 0.05^c^	0.52 ± 0.05^d^	1.51 ± 0.05^d^	5.97 ± 0.01^c^	1.93 ± 0.01^c^	3.95 ± 0.05^d^
S^−^	1.32 ± 0.02^ab^	0.76 ± 0.02^c^	2.08 ± 0.04^c^	5.02 ± 0.02^e^	1.74 ± 0.02^c^	2.41 ± 0.01^e^
S^−^ + NaHCO_3_	1.41 ± 0.09^a^	1.01 ± 0.03^b^	2.42 ± 0.13^b^	5.62 ± 0.10^d^	1.41 ± 0.06^d^	2.32 ± 0.01^e^

All values are expressed as Mean ± SD (n = 3). Values with different letters represent significantly differ at p < 0.05 between the groups. Total Nutrient (TN); Nitrate deficit (N^−^); Nitrate deficit with bicarbonate (N^−^ + NaHCO_3_); Phosphate deficit (P^−^); Phosphate deficit with bicarbonate (P− + NaHCO_3_); Sulphate deficit (S^−^); Sulphate deficit with bicarbonate (S^−^ + NaHCO_3_). Chlorophyll a (Chl-a); Chlorophyll b (Chl-b); Chlorophyll a + b (Chl a + b); Carotenoid (Car); Chlorophyll a/b (Chl a/b); Carotenoid/Chlorophyll a + b (Car/Chl a + b).

### Carbohydrate, lipid and protein content

**Figure 2:**
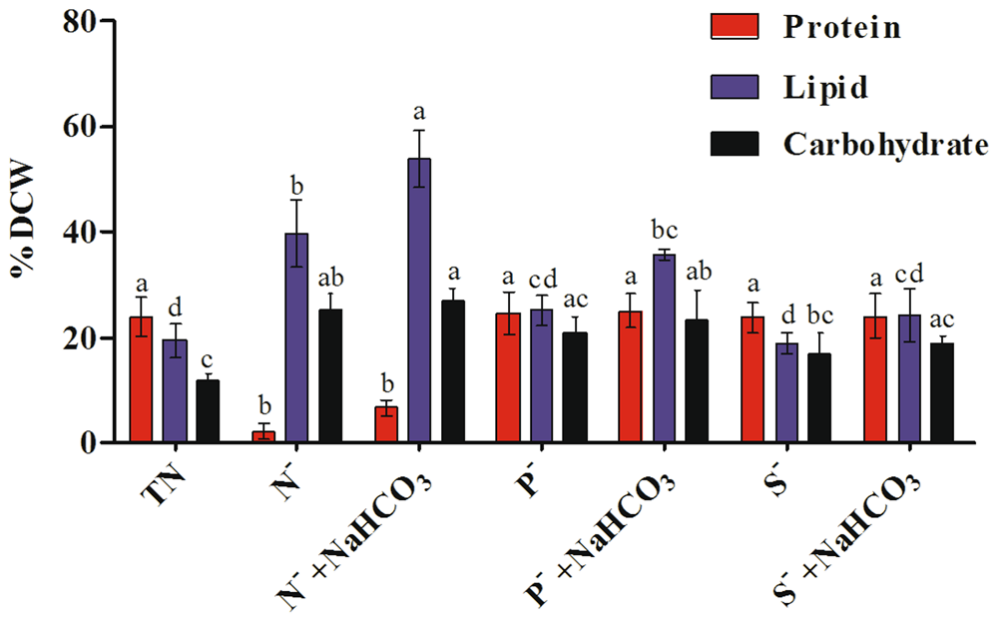
Effect of bicarbonate on protein, carbohydrate and lipid content of dry cell weight (DCW) of *D. salina* under nutrient deficit conditions. All values are expressed as Mean ± SD (n = 3). Values with different letters represent significantly differ at p < 0.05 between the groups. Total Nutrient (TN); Nitrate deficit (N^−^); Nitrate deficit with bicarbonate (N^−^ + NaHCO_3_); Phosphate deficit (P^−^); Phosphate deficit with bicarbonate (P^−^ + NaHCO_3_); Sulphate deficit (S^−^); Sulphate deficit with bicarbonate (S^−^ + NaHCO_3_).

The aim of the study was to investigate the effect of sodium bicarbonate on carbohydrate and lipid content of *D. salina* during nutrient deficit conditions (Fig. 2). The carbohydrate content of *D. salina* was found to be quite higher in bicarbonate supplemented *Dunaliella* cultures compared to nutrient deficient cultures without bicarbonate. Highest carbohydrate content was observed in N^−^ + NaHCO_3_ (27.1 ± 2.31%) followed by P^−^ + NaHCO_3_ (23.3 ± 3.11%) and S^−^ + NaHCO_3_ (19.1 ± 1.09%), respectively. The total lipid content increased from 19.5 ± 3.21 to 53.9 ± 5.32% under N^−^ + NaHCO_3_ compared to N^−^ cultures (39.8 ± 6.32%). Followed by P^−^ culture with and without bicarbonate also showed significant increase in lipid content (25.2 ± 2.98 and 35.7 ± 1.03%). In case of S^−^ and S^−^ + NaHCO_3_, no significant change in the level of lipid contents was observed. Among all macronutrient stresses, nitrogen stress amended with bicarbonate was found to be the ideal method for production of algal lipids, as it resulted in comparatively lower biomass with high carbohydrate and lipid contents in *D. salina*.

Nitrogen is one of the essential components required for synthesis of proteins, and complete removal of nitrate from microalgae cultures led to significant reduction in the protein content from 24.1 ± 3.57% to 2.3 ± 1.62% (crude protein) whereas in bicarbonate amended cultures, recovery of protein synthesis occurred which was indicated by an increase of up to 6.8 ± 1.52% of the crude proteins. P^−^ and S^−^ cultures with or without bicarbonate did not have any significant effect on the protein content of *D. salina* compared to the standard control with adequate nutrients.

### β-carotene and lutein content

**Figure 3:**
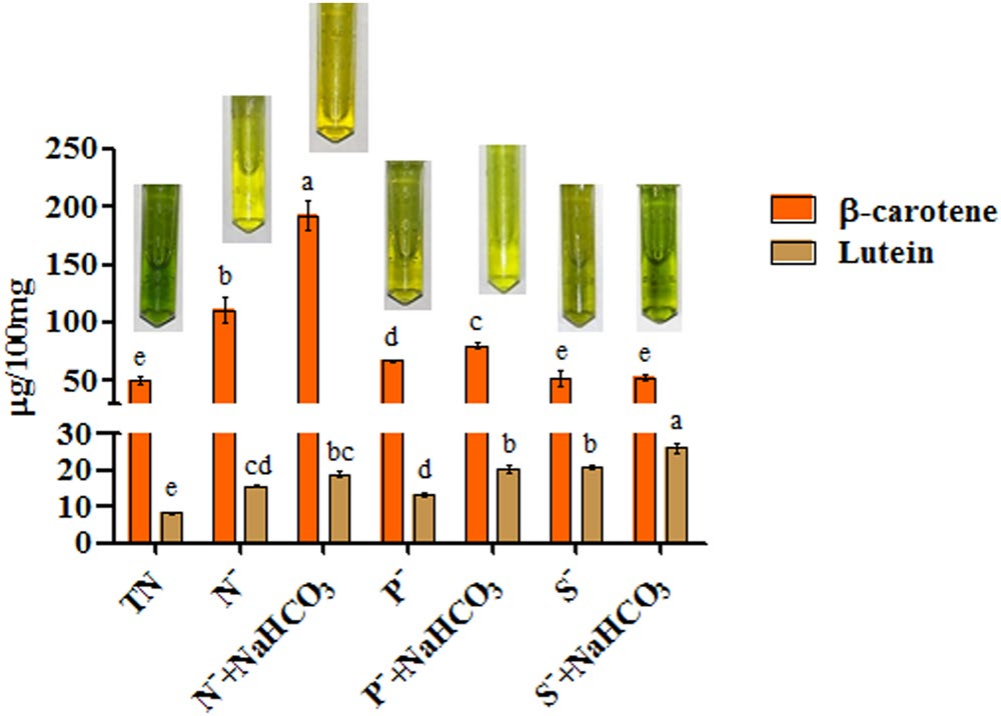
Effect of bicarbonate on β-carotene and lutein of *D. salina* under nutrient deficit conditions. All values are expressed as Mean ± SD (n = 3). Values with different letters represent significantly differ at p < 0.05 between the groups. Total Nutrient (TN); Nitrate deficit (N^−^); Nitrate deficit with bicarbonate (N^−^ + NaHCO_3_); Phosphate deficit (P^−^); Phosphate deficit with bicarbonate (P^−^ + NaHCO_3_); Sulphate deficit (S^−^); Sulphate deficit with bicarbonate (S^−^ + NaHCO_3_).

Figure S1 (Supplementary Material), depicts the chromatograph of individual carotenoid standards (lutein and β-carotene), *Dunaliella* cells were grown under total nutrient and nutrient deficit medium with or without sodium bicarbonate (Figs S2 and S3 of Supplementary Material). The effect of bicarbonate on β-carotene and lutein of *D. salina* upon macronutrient stressed cultures was analyzed by HPLC (Fig. 3). β-carotene and lutein content significantly increased in nutrient stressed cultures amended with bicarbonate compared to stressed cultures without bicarbonate. Highest accumulation of β-carotene was found in N^−^ + NaHCO_3_ (192.8 ± 2.11 µg/100 mg) compared to P^−^ + NaHCO_3_ (79.9 ± 4.92 µg/100 mg) and S^−^ + NaHCO_3_ (53.1 ± 1.01 µg/100 mg) cultures. Lutein accumulation was found to be significantly higher in S^−^ + NaHCO_3_ (26.1 ± 2.12 µg/100 mg) and S^−^ (21.0 ± 1.23 µg/100 mg) cultures compared to other macronutrient depleted conditions.

### Lipids based on FAMEs

As shown in Fig. 2, the total lipid content was found to be high in both nutrient deficit cultures with and without bicarbonate compared to total nutrient. Thus, we determined the relative content of FAMEs in the experimental conditions, using GC-MS (Table S1 of Supplementary Material). Maximum increase in fatty acid accumulation was achieved in N^−^ + NaHCO_3_ treatment (Tetradecanoic acid, 10,13-diethyl, methyl ester – 28.92 ± 0.29% and methyl 16-methyl-heptadecanoate – 30.92 ± 1.11%) in comparison to N^−^ culture (Tetradecanoic acid, 10,13-diethyl, methyl ester – 22.41 ± 1.89% and methyl 16-methyl-heptadecanoate – 24.72 ± 2.31%) followed by P^−^ + NaHCO_3_ (21.4 ± 0.06 and 21.00 ± 0.97%) and S^−^ + NaHCO_3_ (17.31 ± 0.09 and 18.9 ± 1.67), which was significantly (two folds) higher that of control culture.

### Nutrient uptake and activities of nutrient assimilatory enzymes

**Figure 4:**
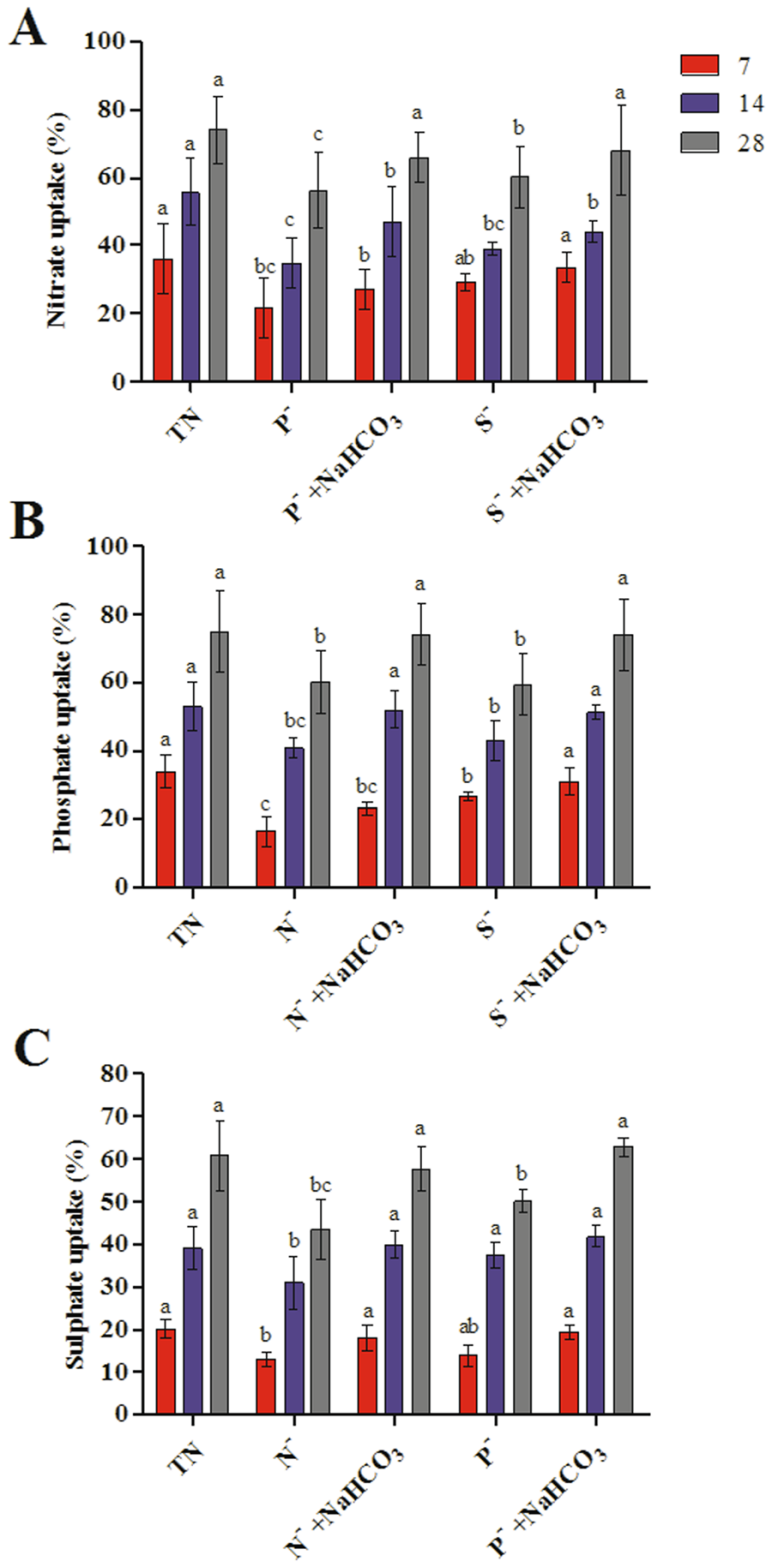
Effect of bicarbonate on nutrient uptake in *Dunaliella* cells grown under nutrient deficit conditions. (**A**) Nitrate uptake, (**B**) Phosphate uptake and (**C**) Sulphate uptake. All values are expressed as Mean ± SD (n = 3). Values with different letters represent significantly differ at p < 0.05 between the groups. Total Nutrient (TN); Nitrate deficit (N^−^); Nitrate deficit with bicarbonate (N^−^ + NaHCO_3_); Phosphate deficit (P^−^); Phosphate deficit with bicarbonate (P^−^ + NaHCO_3_); Sulphate deficit (S^−^); Sulphate deficit with bicarbonate (S^−^ + NaHCO_3_).

**Figure 5:**
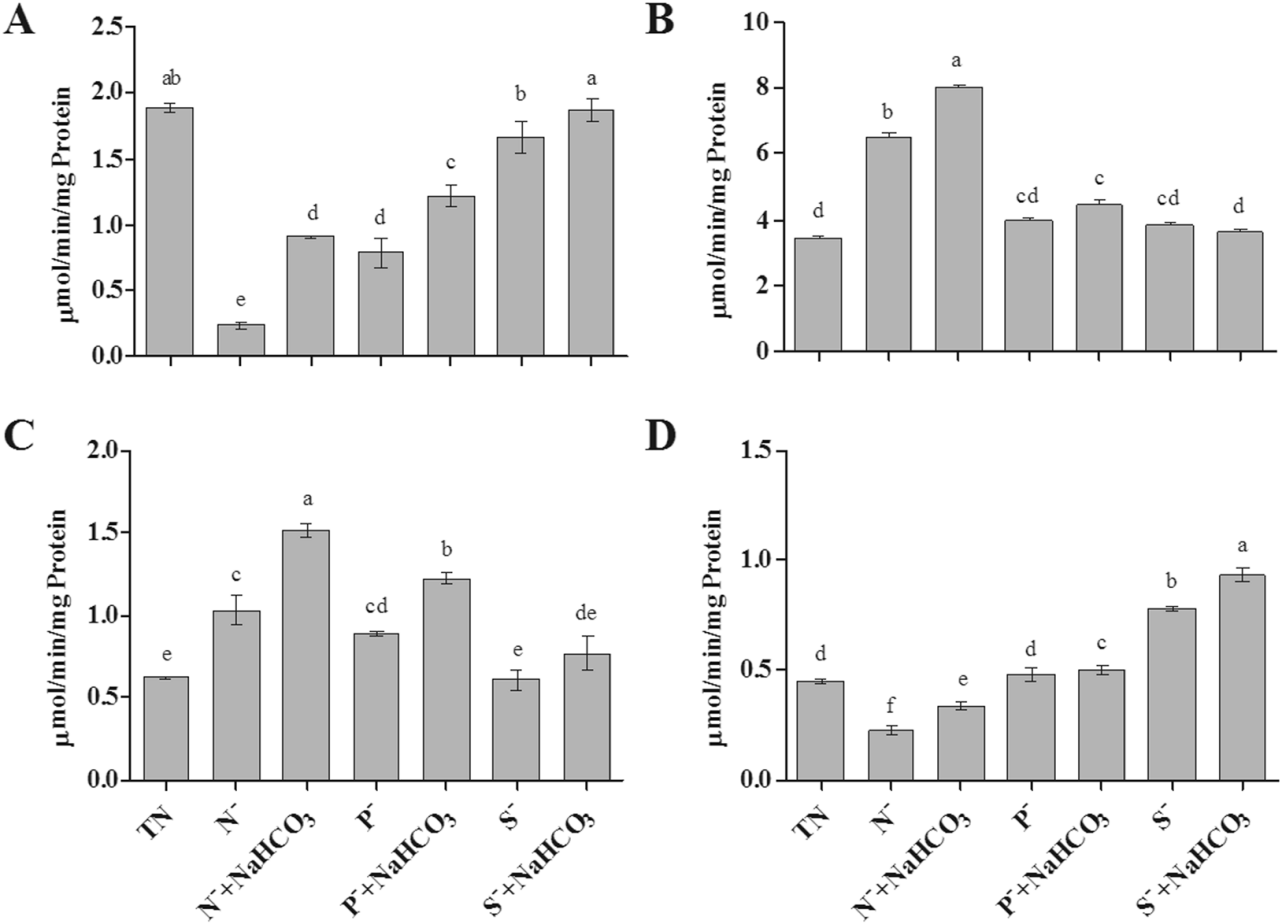
Changes in nutrient assimilatory enzymes nitrate reducatse (**A**), acid phosphatase (**B**), alkaline phosphatase (**C**) and ATP-sulfurylase (**D**) activity of *D. salina* with bicarbonate under nutrient deficit conditions. All values are expressed as Mean ± SD (n = 3). Values with different letters represent significantly differ at p < 0.05 between the groups. Total Nutrient (TN); Nitrate deficit (N^−^); Nitrate deficit with bicarbonate (N^−^ + NaHCO_3_); Phosphate deficit (P^−^); Phosphate deficit with bicarbonate (P^−^ + NaHCO_3_); Sulphate deficit (S^−^); Sulphate deficit with bicarbonate (S^−^ + NaHCO_3_).

Figure 4 shows the effect of bicarbonate amendment on nutrient uptake during nutrient deficit conditions. Assimilation of nutrients was significantly increased by the addition of bicarbonate to the nutrient deficit growth medium. *Dunaliella* cells were grown in total nutrient medium consumed upto 73.90 ± 9.90% of nitrate where cultures grown under phosphate and sulphate deficit medium showed decrease in nitrate uptake of 56.21 ± 10.88% and 60.11 ± 9.10%, respectively (Fig. 4A). In bicarbonate supplemented medium, nitrate uptake was significantly increased in nutrient deficit cultures (P^−^ + NaHCO_3_ – 65.88 ± 7.46% and S^−^ + NaHCO_3_ – 67.94 ± 13.02%). NR activity was significantly reduced during stress conditions. The addition of bicarbonate in nitrate and sulphate deficit cultures resulted in 1.22 ± 0.09 µmol/min/mg protein and 1.87 ± 0.08 µmol/min/mg protein, which was higher than that of nutrient deficit cultures without bicarbonate. Similarly, a decrease in NR activity in N^−^ culture was observed. However, in case of N^−^ + NaHCO_3_, NR activity was found to increase up to 3.8 fold (Fig. 5A).

Addition of bicarbonate resulted in 74.02 ± 9.22% and 74.10 ± 10.73% phosphate uptake in nitrate and sulphate deficit cultures, whereas we observed a decrease in P uptake in nitrate and sulphate deficit cultures without bicarbonate (Fig. 4B). This result shows the significant increase in the AP and ALP enzyme activity in bicarbonate supplemented cultures (Fig. 5B and C). Similarly, assimilation rate of sulphate was also increased in bicarbonate added nutrient deficit medium compared to nutrient deficit medium. Maximum percentage of sulphate uptake was observed in N^−^ + NaHCO_3_ (57.90 ± 5.22%) and P^−^ + NaHCO_3_ (62.97 ± 2.22%) which was higher than that of nitrate and phosphate deficit medium without bicarbonate (Fig. 4C). The activity of ATPS was also found to be increased in bicarbonate supplemented to the medium. Algae grown in S^−^ and S^−^ + NaHCO_3_ showed significant increase in ATPS activity compared with other nutrient deficit conditions (Fig. 5D).

### Activity of total carbonic anhydrase upon addition of bicarbonate

**Figure 6:**
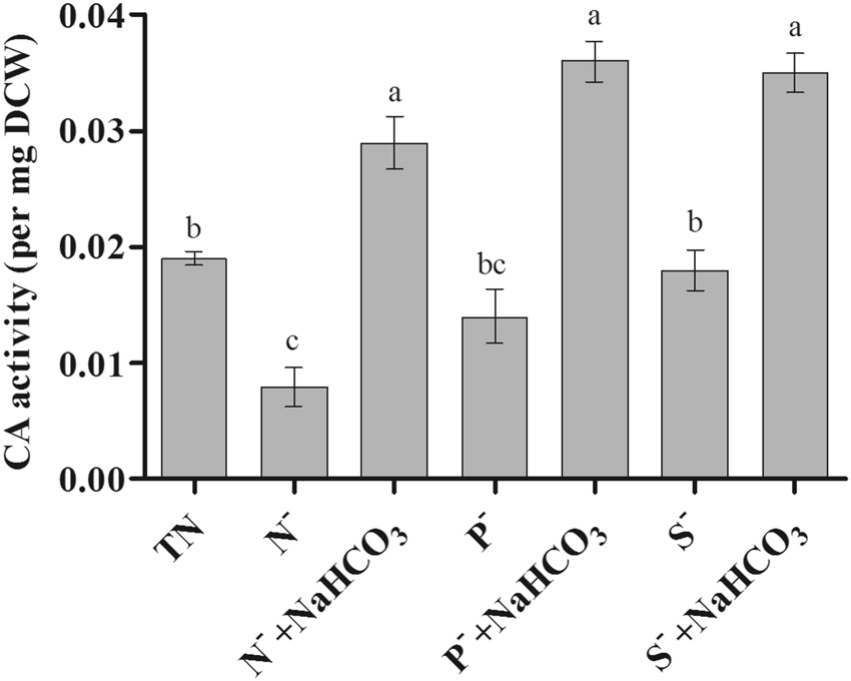
Changes in carbonic anhydrase activity of *D. salina* with bicarbonate under nutrient deficit conditions. All values are expressed as Mean ± SD (n = 3). Values with different letters represent significantly differ at p < 0.05 between the groups. Total Nutrient (TN); Nitrate deficit (N^−^); Nitrate deficit with bicarbonate (N^−^ + NaHCO_3_); Phosphate deficit (P^−^); Phosphate deficit with bicarbonate (P^−^ + NaHCO_3_); Sulphate deficit (S^−^); Sulphate deficit with bicarbonate (S^−^ + NaHCO_3_).

In the present study, the effect of bicarbonate amendment on CA activity of *D.salina* under nutrient deficit conditions was measured as shown in Fig. 6. CA activity was decreased significantly druing N^−^ and P^−^ conditions, whereas S^−^ there is no significant decrease in the CA activity compared to TN conditions. Higher enzymatic activity of CA was observed in nutrient deficient medium amended with bicarbonate (N^−^ + NaHCO_3_ – 0.029 ± 0.004/mg DCW, P^−^ + NaHCO_3_ – 0.036 ± 0.003/mg DCW and S^−^ + NaHCO_3_ – 0.035 ± 0.003/mg DCW), as compared to total nutrient medium (0.019 ± 0.001/mg DCW)

### ROS levels and antioxidant enzymes

**Figure 7:**
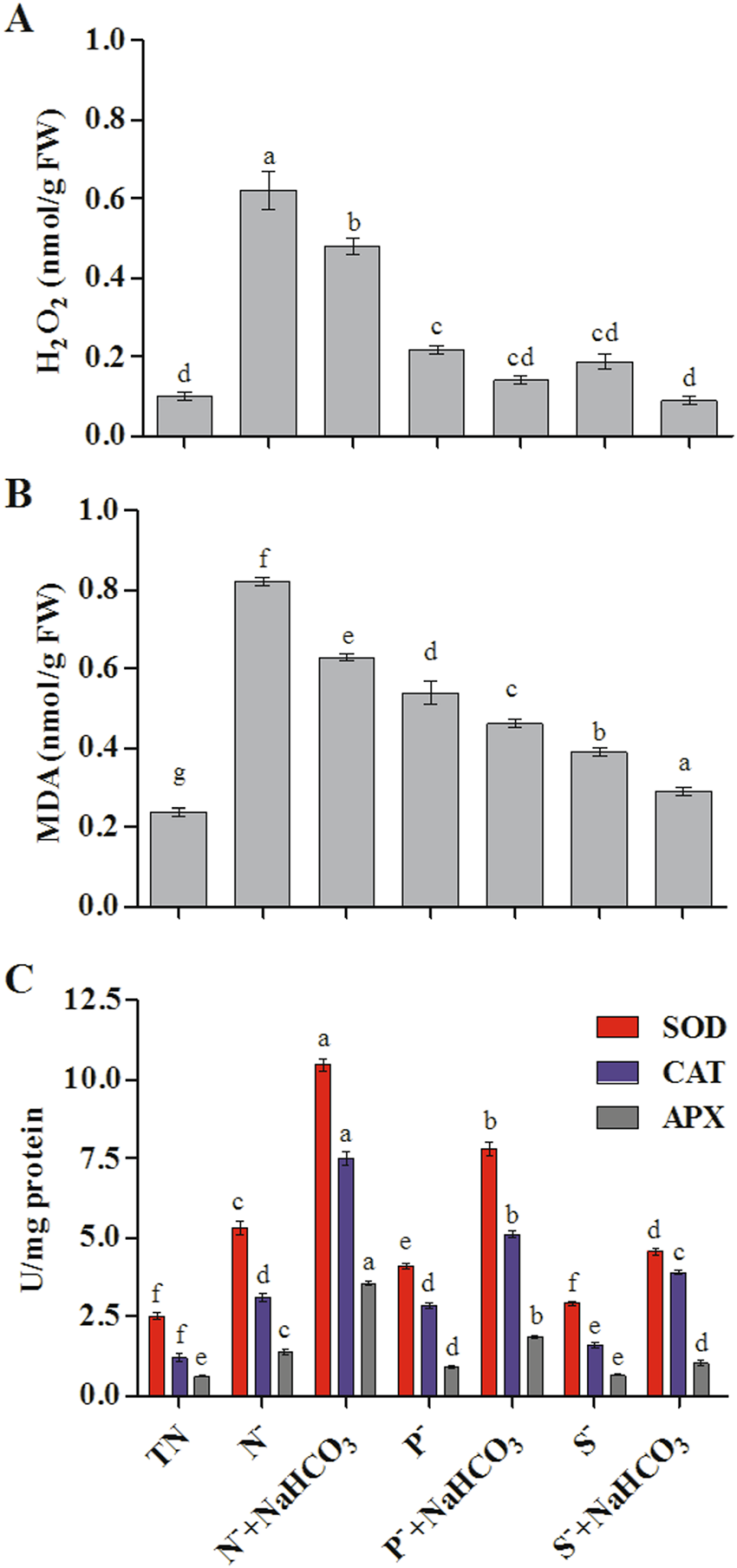
Production of ROS (**A**), lipid peroxidation (**B**) and antioxidant acitivities of SOD, CAT and APX (**C**) of *D. salina* with bicarbonate under nutrient deficit conditions. All values are expressed as Mean ± SD (n = 3). Values with different letters represent significantly differ at p < 0.05 between the groups. Total Nutrient (TN); Nitrate deficit (N^−^); Nitrate deficit with bicarbonate (N^−^ + NaHCO_3_); Phosphate deficit (P^−^); Phosphate deficit with bicarbonate (P^−^ + NaHCO_3_); Sulphate deficit (S^−^); Sulphate deficit with bicarbonate (S^−^ + NaHCO_3_).

The concentration of H_2_O_2_, was increased under nutrient depleted conditions in comparison with total nutrient culture (Fig. 7A). All nutrient deficit algal cultures that were amended with bicarbonate showed reduction in the level of H_2_O_2_ radicals compared to cultures without bicarbonate (0.62 to 0.55 nmol/g FW (N^−^ + NaHCO_3_), 0.22 to 0.20 nmol/g FW (P^−^ + NaHCO_3_) and 0.19 to 0.14 nmol/g FW (S^−^ + NaHCO_3_)). Figure 7B, MDA content was decreased in *D. salina* cultures grown under nutrient deficit conditions amended with bicarbonate than that of the cultures without bicarbonate from 0.82 to 0.63 nmol/g FW (N^−^ + NaHCO_3_), 0.57 to 0.49 nmol/g FW (P^−^ + NaHCO_3_) and 0.39 to 0.31 nmol/g FW (S^−^ + NaHCO_3_). Activities of antioxidant enzymes in *D. salina* during nutrient stress in the presence or absence of bicarbonate showed that the activities of SOD, CAT and APX were enhanced in nutrient stress cultures amended with bicarbonate (N^−^ + NaHCO_3_ − 10.45 ± 0.19, P^−^ + NaHCO_3_ – 7.82 ± 0.20 and S^−^ + NaHCO_3_ − 4.56 ± 0.10 U/mg protein, respectively) when compared to nutrient stress cultures without bicarbonate (Fig. 7C).

## Discussion

Cultivation conditions including pH, light intensity, temperature, nutrient depletion or repletion, osmotic stress and other stresses significantly affect the growth and biochemical composition of microalgae^17–19^. Especially, during macronutrient limitation or deficit conditions, microalga accumulate high quantities of lipids and carotenoids^3,20^. Maintaining high growth rate and biomass productivity is often necessary for good product yield, which has been a major problem since upholding optimum culture condition, escalates the cost of production^21^. In microalgae, supplementation of inorganic carbon is one of the important factor for influencing growth, photosynthesis process and generation of biomass with desired products. Many microalgae have the ability to uptake dissolved inorganic carbon (DIC) from the surrounding for active photosynthesis by CO_2_ concentrating mechanism^22^. In various microalgae addition of sodium bicarbonate to the culture medium resulted in high pH as well as high DIC in the growth medium which increased their growth and higher TAG accumulation^6,7^. However, the effectiveness of bicarbonate addition in macronutrient deficit cultures like N-, P- and S- in marine microalgae is still unknown. From our previous report, addition of dissolved inorganic carbon especially, sodium bicarbonate enhances the biomass of *D. salina* as well as its chlorophyll, β-carotene, lipids and fatty acid content^23^. Growth and biomass of *Dunaliella* were high in nitrate and phosphate deficit cultures amended with bicarbonate, whereas in case of S^−^ condition, the growth and biomass of *D. salina* was not significantly affected compared to standard control cultures. These results indicate that nitrate and phosphate are essential for growth and homeostasis in the microalga, *D. salina*. Similarly, several reports also revealed the increase in growth and biomass of microalgae and diatoms cultured in bicarbonate amended medium^8,15,16,24^.

The pH of the experimental growth medium with bicarbonate indicated a pH of 9.4. This observation can lead to the understanding that HCO_3_^−^ is utilized as a carbon source to avoid the limitations of using carbon via carbon concentrating mechanisms^25^. Similar results have been observed in several other microalgal species where a high pH and nutrients deficit condition causes triacylglycerol (TAG) accumulation independently^6,7^. Peng and his co-workers^26^ proved that combination of 160 mM NaHCO_3_ and pH 9.5 showed maximum microalgal growth when compared with 160 mM/pH 8.5 and 160 mM/pH 7.5. In our results, all nutrient deficit cultures showed decreased photosynthetic efficiency compared to control containing total nutrients. A previous report from Gao *et al*.^20^ also showed reduction of efficiency of photosynthesis in *D. salina* from seawater upon complete nutrient deprivation. Among our applied stress conditions, N^−^ cultures showed the lowest photosynthetic activity. Therefore, nitrate removal majorly affects protein synthesis (including those of the reaction centers) and results in lowering of chlorophyll content which, in turn, reduces the efficiency of PSII photosystem. Carbon dioxide is one of the major factors that influence the photosynthesis process of microalgae. Addition of sodium bicarbonate improved photosynthesis efficiency in all the nutrient deficit cultures. Similar results were observed in *C. pyrenoidosa* and *S. obliquus* when exposed to higher concentration of CO_2_ (186 µM)^27^.

Carotenoids and chlorophyll are indicators of stress in microalgae during exposure to various biotic and abiotic stress conditions. Photosynthetic pigments such as chlorophyll and carotenoid were quite higher in bicarbonate supplemented medium when compared to nutrient deficit medium. These changes may lead to the modifications in the structure of photosynthetic apparatus i.e. loss of PSII reaction center proteins in marine microalgae under nutrient limited conditions^5^. Nitrate deficit medium showed reduction of chlorophyll content in microalgae. This indicates that chlorophyll is a nitrogenous compound which is strongly influenced by nitrate content in the growth medium. Our results also corroborate with these findings. Nutritional starvation has often been proposed as a potential strategy to enhance the biochemical composition of microalgae^21^. In our study, removal of macronutrients especially nitrogen resulted in significant decrease in crude protein, but the addition of bicarbonate aided in the recovery of protein synthesis during stress conditions. Similarly, Pancha *et al*.^28^, in their experiments showed a steady decrease in protein content under nutrient starved conditions. Addition of bicarbonate has been shown to marginally increase the protein content of freshwater alga *Scenedesmus* sp. CCNM1077^8^. Other studies involving phosphate and sulphate limiting conditions have reported no significant increase of protein content in microalgae^3,8^. In nutrient starved algal cultures, lipids and carbohydrates are the most preferred storage products due to their hydrophobic and reduced state nature in cells^28^. However, the effect of addition of bicarbonate on algal lipids under deficit conditions has not been studied in *Dunaliella* sp. The present study revealed that amendment of bicarbonate significantly enhanced the carbohydrate and lipid content compared to microalgae grown under nutrient deficit condition. Other studies including, Guiheneuf and Stengel,^29^, Mondal *et al*.^9^ and Pancha *et al*.^8^, showed that addition of bicarbonate in nitrate deficit medium to improve the carbohydrate, lipid and TAG content in the microalgae.

An adequate supply of inorganic carbon is also essential to maintain photosynthetic, carbon fixation and the growth of microalgae^29^. Microalgae that grow photoautotrophically use inorganic carbon to synthesis, *de novo* to produce their own organic carbon compounds. Many microalgae and diatoms have been investigated to study the effect of sodium bicarbonate and results indicate sudden ceasing in cellular replication and rapid accumulation of fatty acid composition such as triacylglycerols and omega-3 fatty acids^29–31^. Saturated fatty acids are produced more predominantly in *Dunaliella* during high CO_2_ conditions than monounsaturated and polyunsaturated fatty acids. This difference in the fatty acid content and composition indicate the activation of fatty acid synthesis *de novo* and inhibition of desaturation as well as elongation of fatty acid^32^. Similar to our results, several findings also indicated that the addition of bicarbonate significantly enhanced the fatty acid profile with higher amount of saturated fatty acids under nutrient starvation conditions in microalgae^6,7,29^.

Enzyme mediated nutrient assimilation from marine environment and variation in the activity of enzymes may directly reflect the nitrate, phosphate and sulphate assimilation process and physiological process of phytoplankton^33^. We observed that the enzyme activity of NR activity was found to be higher in N^−^ + NaHCO_3_ when compared to N^−^. Changes in the intracellular ATP:NADP + /NADPH (Adenosine triphosphate:Nicotinamide adenine dinucleotide phosphate) ratio, which could affect the NR activity that is required to convert nitrate into nitrite in the presence of NADPH as reducing agent^34^. Under normal CO_2_ conditions, *Chlamydomonas* require higher ratio of ATP:NADPH ratio for CO_2_ assimilation. If the algae was grown in elevated CO_2_ conditions, which would then require a low ratio of ATP:NADPH, the excess NADPH could stimulate the NR activity^35^. Giordano *et al*.^36^, also reported another possible reason for stimulation of the NR activity under high CO_2_, where such conditions are controlled by the redox state of the plastoquinone pool in microalgae and where the NR activity is stimulated by a reduced plastoquinone pool. We also found that AP and ALP activity were significantly higher in bicarbonate amended culture than those cultures without bicarbonate. Activity of AP has been reported to be generally higher compared to ALP in other algae including *Chlorella*, *Chlamydomonas* and *Scenedesmus* sp. CCNM 1077^8,37^. This is because; AP is the first enzyme that helps to liberate intracellular phosphate, whereas ALP helps to remove dissolved organic phosphate from the medium when the intracellular pool is exhausted^38^. Rouco *et al*.^39^ studied the activity of APase in *Emiliania huxleyi* (NZEH) and proved that increase in the APase activity was a clear response to CO_2_ partial pressure, and its maximum activity was observed at 561 microatm CO_2_ and higher pH (8.18) was also responsible for the enhanced activity of APase. The activity of AP and ALP was significantly low in phosphate deficit cultures compared to nitrate and sulphate deficit cultures. This clearly suggests that induction of AP and ALP enzyme depends upon the presence of phosphate content in the medium. ATPS is the first enzyme involved in the sulfate reduction pathway and increase in its activity indicates the stimulation of sulfate assimilation. Similar to our observation, Giordano *et al*.^40^, also proposed that in *D. salina* sulfur limiting conditions resulted in an increase of ATPS activity whereas decreased the NR activity. This suggests that increase of ATPS is an adaptation to reduce availability of sulfur by accumulation of the enzyme for effective use of the substrates in microalgae.

During the culture conditions, enhancing the catalytic activity of CA, which faciliates the rapid conversion of HCO_3_^−^ to CO_2_, could effectivley meet the demand for carbon needed for photosynthesis and increase the growth of microalgae^15^. Our results indicate that the supplementation of bicarbonate enhanced the CA activity under nutrient deplete conditions (Fig. 4). To our knowledge, a few studies have focused on the effect of bicarbonate on the induction of enzymes invovled in carbon concentarting mechanism, such as CA^41,42^. Their results show that adequate bicarbonate addition could enhance CA activity whereas, when gaseous CO_2_ its activity was found to be decreased^43^. Possibly, gaseous CO_2_ can form a CO_2_ gradient through simple diffusion of CO_2_ around the cell; however, bicarbonate salts need conversion through CA activity to form a CO_2_ gradient^42^. A recent study also reported that addition of bicarbonate (160 mM) alleviates oxygen stress on *Neochloris oleoabundans* as well as enhances the activity of CA when compared to treatment without bicarbonate^15^.

β-carotene and lutein are the two major carotenoids that accumulated largely in *Dunaliella* sp. during the nutritional stress conditions. Generally, nutrient deficiency slows down the photosynthetic carbon fixation and results in the accumulation of lipids along with carotenoids in microalgae^3^. These carotenoids play an important role in the eukaryotic cells in response to ROS produced during nutritional stress i.e. removal of nutrients, low or high temperature, osmotic stress, irradiation stress^5,44^. In our experiments, addition of bicarbonate was found to enhance the accumulation of β-carotene and lutein was observed in nutrient deficit *Dunaliella* cultures. Studies have shown that high CO_2_ concentration can induce the transition state of photosynthetic apparatus from I to II, which has been suggested to increase cyclic electron transport and to generate the surplus amounts of ATP necessary for support of pH homeostasis in the algal cell^45,46^. As a result, carotenoid pigments were binding to the L1 site of antenna proteins^47^, it is reasonable that the carotenoid content increased slightly with increasing CO_2_ concentration. In *D. salina*, next to β-carotene, lutein is also an important carotenoid that protects cell from damage incurred by reactive oxygen species (ROS) generated during stressful conditions^48^. Insterestingly, *D. salina* cells subjected to S^−^ and S^−^ + NaHCO_3_ showed significant increase in lutein content compared to nitrate and phosphate deficit conditions. Recently, Lv *et al*.^5^ also reported that higher accumulation of lutein in *D. salina* occurs during sulphate starvation compared to other starvation conditions and expression analysis indicates that the regulation of lycopene *ε*-cyclase and lycopene *β*-cyclase was influenced differently by sulfur availability.

Organisms can activate several defense mechanisms for scavenging ROS, but under unfavorable conditions out-paced ROS leads to cell damage^4^. It has been suggested that excess ROS could be involved in massive accumulation of carotenoids and modulate the antioxidant enzyme levels in *D. salina* under macronutrient stress conditions^5^. This may be the first report to investigate the regulatory effect of sodium bicarbonate on ROS and antioxidant enzymes activities in response to three different nutrient stresses. Hydroxyl radicals such as OH^−^, and hydrogen peroxide H_2_O_2_ are highly toxic forms of reactive oxygen, and also the intermediate molecules produced predominantly in microalgae during stress conditions and it has sufficient reactivity to initiate membrane peroxidation by liberation of MDA as an end product^49^. MDA is an end product of lipid peroxidation usually released as an indicator in microalgae under stress condition^50^. In *Dunaliella*, the addition of bicarbonate reduced the generation of ROS and lipid peroxidation during nutrient deficient conditions. These results were corroborates with earlier finding by Peng *et al*.^15^ who suggested that the effect of bicarbonate or dissolved inorganic carbon on oxygen stress may be attributed to the inhibition of ROS, which was clearly demonstrated by the decline in ROS levels in the media containing bicarbonate. Earlier findings also proved that addition of sodium bicarbonate enhances the cell growth in many algal species by reducing the oxidative stress^7,8,15^. In our results, addition of bicarbonate was found to increase the antioxidant enzymes activities, which was quite consistent with results of Peng *et al*.^16^ and Fawzy *et al*.^16^.

In this study, addition of bicarbonate showed ameliorating effects by enhancing the biomass and biochemical composition of *D. salina* against oxidative stress induced by macronutrient deficiency. Among these three macronutrient stresses, nitrate depletion coupled with bicarbonate supplementation was the best strategy to improve both carotenoid and lipid content simultaneously with minimal loss of biomass compared to other nutrient stress treatments. Thus, microalgal cultures amended with bicarbonate can improve synthesis of industrially important products by overcoming the pitfalls of the conventional method of imposing stress to improve productivity. Further, this amelioration of stress by bicarbonate employs rescuing cellular redox homeostasis as a major strategy to sustain growth in the microalgae *D. salina*.

## Material and Methods

### Strain and Culture conditions

*Dunaliella salina* (V-101) was obtained from Centre for Advanced Studies in Botany, University of Madras, Chennai, India and axenic algal culture was maintained in De Walne’s medium. From our previous study^23^, we were aware that 100 mM concentration of sodium bicarbonate was the threshold to enhance the biochemical content (carotenoids and fatty acids) as well as the growth and biomass of *Dunaliella* sp. Thus, the present study is extended to investigate the effect of sodium bicarbonate on biochemical and antioxidant status of *D. salina* under three different nutrient stresses. For nitrate stress, complete removal of sodium nitrate and ammonium molybdate from the media was carried out; phosphate stress involved complete removal of sodium dihydrogen phosphate and for sulphate stress; copper sulphate from the medium was substituted with cupric chloride.

All the experiments were conducted in triplicate for each nutrient stress (N^−^, P^−^ and S^−^) and sodium bicarbonate amended treatments, in one liter flasks containing 500 mL of culture medium inoculated with 10% of actively growing algal culture. Batch cultures were incubated at 24 ± 0.2 °C in a growth chamber with illumination of 50 μmol m^−2^ s ^−1^ under a 16:8-h photoperiod. The cultures were taken out and shaken manually on a day-to-day basis.

### Biomass and biochemical composition analysis

#### Estimation of growth, biomass and photosynthetic capacity

Growth of *D. salina* and pH of the medium was monitored at regular intervals (every 7 days) by measuring optical density at 750 nm using a Gene Quant 1300 UV spectrophotometer. For dry mass calculation, twenty milliliters of cultures were harvested by centrifugation at 8000 rpm for 5 min. The pellets were washed gently twice with 1.5 mL of sterile milliQ water to remove the culture medium containing salts. The sample was transferred into pre-weighed 1.5 mL eppendrof tubes. Cells were again centrifuged at 2000 rpm for 5 min, and the supernatant was discarded. The tubes were dried in a hot air oven at 80 °C for 48 hrs. The dry weight of biomass was estimated by gravimetrically. The pH of the medium was monitored over a period of 28 days, using Thermo Scientific Eco Tester pH1.

The maximum photosynthesis performance of PSII (F_*v*_/F_m_) of algal samples was measured using PAM AquaPen fluorometer. The samples were kept in the dark for 15 min before measurement. The original fluorescence (F_0_) was determined by measuring the light and a maximum fluorescence (F_m_) measured after applying a saturation pulse to the samples in dark. The effective PSII quantum yield was calculated as

1 $${{\rm{F}}}_{v}/{{\rm{F}}}_{{\rm{m}}}=({{\rm{F}}}_{{\rm{m}}}-{{\rm{F}}}_{0})/{{{\rm{F}}}_{{\rm{m}}}}^{4}.$$

### Determination of photosynthetic pigments

For determining the amount of photosynthetic pigments, *Dunaliella* cells were harvested and the cell contents were extracted with 100% acetone under dark at room temperature. The absorbance of chlorophyll a, b and carotenoids content was measured at 650 nm, 665 nm and 470 nm respectively, using GeneQuant 1300 UV spectrophotometer^51^.

2 $${\rm{Chlorophyll}}\,a({\rm{\mu }}g/{\rm{mL}})=11.75({{\rm{A}}}_{662})-2.35({{\rm{A}}}_{645})$$

3 $${\rm{Chlorophyll}}\,b({\rm{\mu }}g/{\rm{mL}})=18.61({{\rm{A}}}_{645})-3.96({{\rm{A}}}_{662})$$

4 $${\rm{Total}}\,{\rm{carotenoids}}({\rm{\mu }}g/{\rm{mL}})=(1000{{\rm{A}}}_{470}-2.270\,{\rm{Chl}}{a}-81.4\,{\rm{Chl}}{b})/198$$

### Estimation of carbohydrates, protein and lipid content

Lyophilized algal cells were extracted using chloroform:methanol (2:1) mixture and total lipid content was estimated by colorimetry using the sulfo-phospho-vanillin method^52^. Total lipid samples were diluted with twice the volume of chloroform:methanol (2:1) for the colorimetric assay. Fifty microlitre aliquots of each diluted samples were directly added into the bottom of the plate followed by incubation at 90 °C until complete evaporation of solvents. After evaporation, 50 µL of concentrated sulphuric acid was added to each well and incubated at 90 °C for 20 min. Background absorbance was recorded at 540 nm, after cooling the plates to room temperature. Aliquots of 25 µL of vanillin-phosphoric acid solution were added to each well and the plate was incubated at room temperature for color development. Then, the microtitre plate was read at 540 nm using Microtitre plate reader, BioTek. Triolein (2 to 120 µg) was used to generate the standard curve for lipid content.

For protein content analysis, total protein was extracted from 5 mg of algal cells^53^ and the total protein present in the supernatant was estimated at 660 nm using Lowry method^54^. Carbohydrate content was measured by acid hydrolysis of lipid according to Wychen and Laurens^55^. Resulting supernatant was used for the estimation of carbohydrate by Phenol-Sulphuric acid method.

### Measurements of β-carotene and lutein content

Lyophilized algal cells were extracted with acetone and concentrations of carotenoids β-carotene and lutein were measured using High performance liquid chromatography, HPLC (Shimadzu LC 20 A equipped with double pump and UV-Vis detector). Each sample was eluted with isocratic solvent acetonitrile:methanol:dichloromethane (70:10:20) at a flow rate of 1.0 mL/min and carotenoids were detected at 470 nm. β-carotene and lutein standards were used to quantify the level of carotenoids present in the each culture^56^.

### Analysis of fatty acid content

Fatty acid methyl esters (FAMEs) were prepared from total lipids as described in Ryckebosch *et al*.^57^ using Gas Chromatography-Mass Spectroscopy (GC-MS). The column was held at an initial temperature of 60 °C for 1 min and ramped to 300 °C at 10 °C/min, and it was then maintained under same conditions for 6 min. The transfer line between GC-MS was kept at 240 °C. Helium gas was used as carrier and column flow was at 0.5 mL min^−1^. The data was recorded and processed with Turbo Mass software (Version 5.4, Perkin Elmer, USA).

### Determination of nutrient uptake and nutrient assimilatory enzymes

At different time points, 10 mL of culture medium was collected from experimental samples and stored at 4 °C for further analysis. For nitrate content, 0.25 mL of culture medium was mixed with 0.8 mL of 5% (w/v) of salicylic acid in concentrated sulphuric acid. After 20 mins of incubation, 8 mL of 2 N sodium hydroxide was added to raise pH of above 12 and allowed the reaction mixture to cool at room temperature. The residual nitrate content of culture was measured at 410 nm^58^. For phosphate content, 2.5 mL of culture medium was added with 400 µL combined ascorbic acid reagent (5 N sulphuric acid, 1.3 g of potassium antimonyl tartrate, 2 g of ammonium molybdate and 1.7 g of ascorbic acid) and incubated the samples for 10 mins. The absorbance was measured at 880 nm^59^. For sulphate content, 1.0 mL of culture medium was added with 0.5 mL of sodium chloride – hydrochloric acid solution (25 g of sodium chloride and concentrated hydrochloric acid). Then, 0.1 g of barium sulphate was added and mixed well. The absorbance of reaction mixture was recorded at 420 nm^60^.

Nitrate reductase (NR) activity was determined by the production of nitrite according to the method of Li *et al*.^61^ while the activities of acid and alkaline phosphatase (AP and ALP) were determined, using *p*-nitrophenylphosphate (*p*-NPP) as substrate following protocol of Lee^38^. The activity was represented by the production of *p*-NP released by *p*-NPP. ATP-sulfurylase (ATPS) activity was measured by coupling the reverse reaction of ATPS with the APS-dependent reduction of NADP catalyzed by hexokinase and glucose-6-P dehydrogenase^62^. Total soluble protein content was measured using bovine serum albumin as a standard^63^.

### Total carbonic anhydrase activity

The total carbonic anhydrase (CA) of *D. salina* was determined according to the protocol of Wilbur and Anderson^64^. One hundred milligrams of microalgal cells were ground in mortar with liquid nitrogen and powdered cells were mixed with 1 mL of chilled assay buffer (50 mM Tris at pH 8.7 containing 5 mM EDTA, 25 mM isoascorbic acid and 25 mM dithiothreitol). Then, 1.5 mL of the mixture was added to a clean dry tube followed by the addition of 2 mL ice-cold CO_2_-saturated water. The total activity of CA enzyme was calculated using the formula,

5 $${\rm{Enzymatic}}\,{\rm{activity}}\,{\rm{of}}\,{\rm{CA}}={{\rm{T}}}_{{\rm{b}}}{{\rm{T}}}_{{\rm{s}}}-1/{\rm{DCW}}$$

where, T_b_ is the time required for the blank to decrease pH by 2 units, T_s_ is the time required for the sample to decrease pH by 2 units and DCW is the dry cell weight of the algal sample.

### ROS and antioxidant capacity

#### Release of H_2_O_2_ (Hydroxyl radical) and Malondialdehyde

Hydrogen peroxide levels were determined as described by Alexieva *et al*.^65^. To algal pellets, 5 mL of 0.1% (w/v) cold TCA (Trichloro acetic acid) was added in an ice bath followed by sonication (twice) for 30 s with 2 min interval. The homogenized mixture was then centrifuged at 12,000 × *g* for 15 min and the resulting supernatant was used for determination of H_2_O_2_ as well as lipid peroxidation levels. The reaction mixture for H_2_O_2_ determination contained 0.5 mL of 100 mM potassium phosphate buffer (pH 7.4), 0.5 mL of the supernatant and 2 mL of 1 M potassium iodide. The mixture was incubated at room temperature for 1 h in dark and their absorbance recorded at 390 nm.

For lipid peroxidation, the formation of thiobarbituric reactive substance (TBA-MDA [Thiobarbituric acid-Malondialdehyde] complex level) in the algal cells was assayed by the method of Heath and Parker^66^. One milliliter of supernatant from microalgae was mixed with 2 mL of reagent (0.5% (w/v) TBA in 20% TCA). The mixture was boiled at 95 °C for 30 min and then the reaction was stopped by incubating in an ice bath. The absorbance of the samples was recorded at 532 and 600 nm after centrifugation at 10,000 × *g* for 10 min. After subtracting the non-specific absorbance at 600 nm, the MDA–TBA complex content was calculated assuming an extinction coefficient (*ε*) of 155 mM^−1^ cm^−1^.

### Intracellular antioxidant enzyme activity

To measure antioxidant activity, algal cells (50 mL) were harvested and ground to powder in liquid nitrogen using motor and pestle. Pulverized samples (0.5 g) were homogenized in 10 mL of solution containing 50 mM of potassium phosphate buffer and 1% (w/v) polyvinylpyrrolidone (pH 7.8) and kept at 4 °C for 10 min. The homogenate was filtered followed by centrifugation at 4,000 × *g* for 15 min at 4 °C. The supernatant was used as the enzyme extracts and was transiently stored at 4 °C before analysis. Total superoxide dismutase (SOD) activity was assayed in terms of reduction in absorbance due to inhibition of the nitro-blue tetrazolium (NBT) photochemical reduction reaction as described by Beauchamp and Fridovich^67^, catalase (CAT) activity was assayed spectrophotometrically by measuring the decrease in absorbance at 240 nm due to H_2_O_2_ decomposition^68^ and ascorbate peroxidase (APX) was assayed by measuring the decrease in absorbance at 290 nm occurring due to oxidation of ascorbic acid to dehydroascorbate according to the method of Chen and Asada^69^.

### Statistical Analysis

All experiments were performed in triplicate. Statistical analysis was performed by one-way ANOVA, followed by Tukey-Kramer (HSD) for comparison of specific treatment using Graph pad Prism version 5.0. The results were considered statistically significant if the *p* value was < 0.05.

## Electronic supplementary material

Supplementary material
