# Supplementary material for: Bicarbonate supplementation enhances growth and biochemical composition of Dunaliella salina V-101 by reducing oxidative stress induced during macronutrient deficit conditions
Source: Sci Rep. 2018 May 3;8:6972. doi: 10.1038/s41598-018-25417-5 (PMC5934444; doi:10.1038/s41598-018-25417-5)
Supplement: Supplementary file 1 — Supplementary material [file 41598_2018_25417_MOESM1_ESM.doc]

**Bicarbonate supplementation enhances growth and biochemical composition of *Dunaliella salina* V-101 by reducing oxidative stress induced during macronutrient deficit conditions**

Ramachandran Srinivasan^a^, Anbazhagan Mageswari^a^, Parthiban Subramanian^b^, Chandrasekaran Suganthi^a^, Amballa Chaitanyakumar^a^, Velmurugan Aswini^a^, Kodiveri Muthukalianan Gothandam^a*^

^a^School of Bio-Sciences and Technology, Vellore Institute of Technology, Vellore – 632 014, Tamil Nadu, India

^b^Department of Agricultural Biotechnology (Metabolic Engineering Division), National Institute of Agricultural Sciences, Rural Development Administration, Jeonju, Republic of Korea


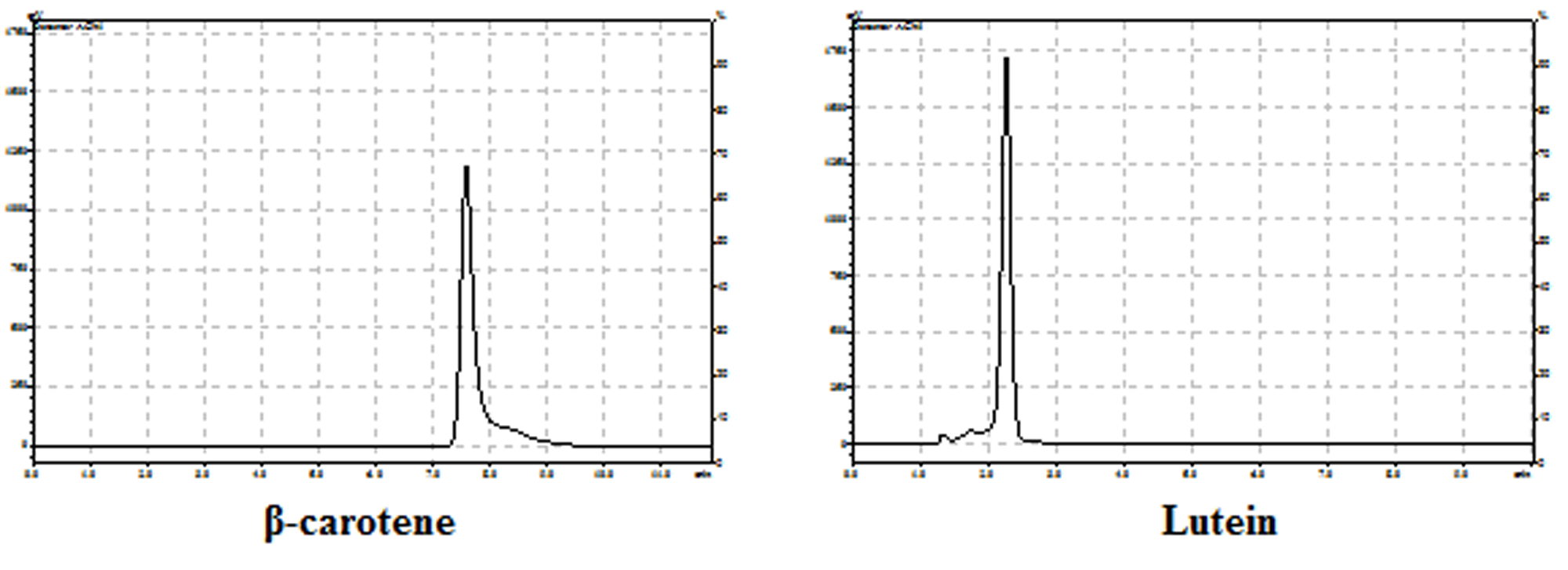


Fig. S1. HPLC chromatograph of carotenoid standards (β-carotene and Lutein).


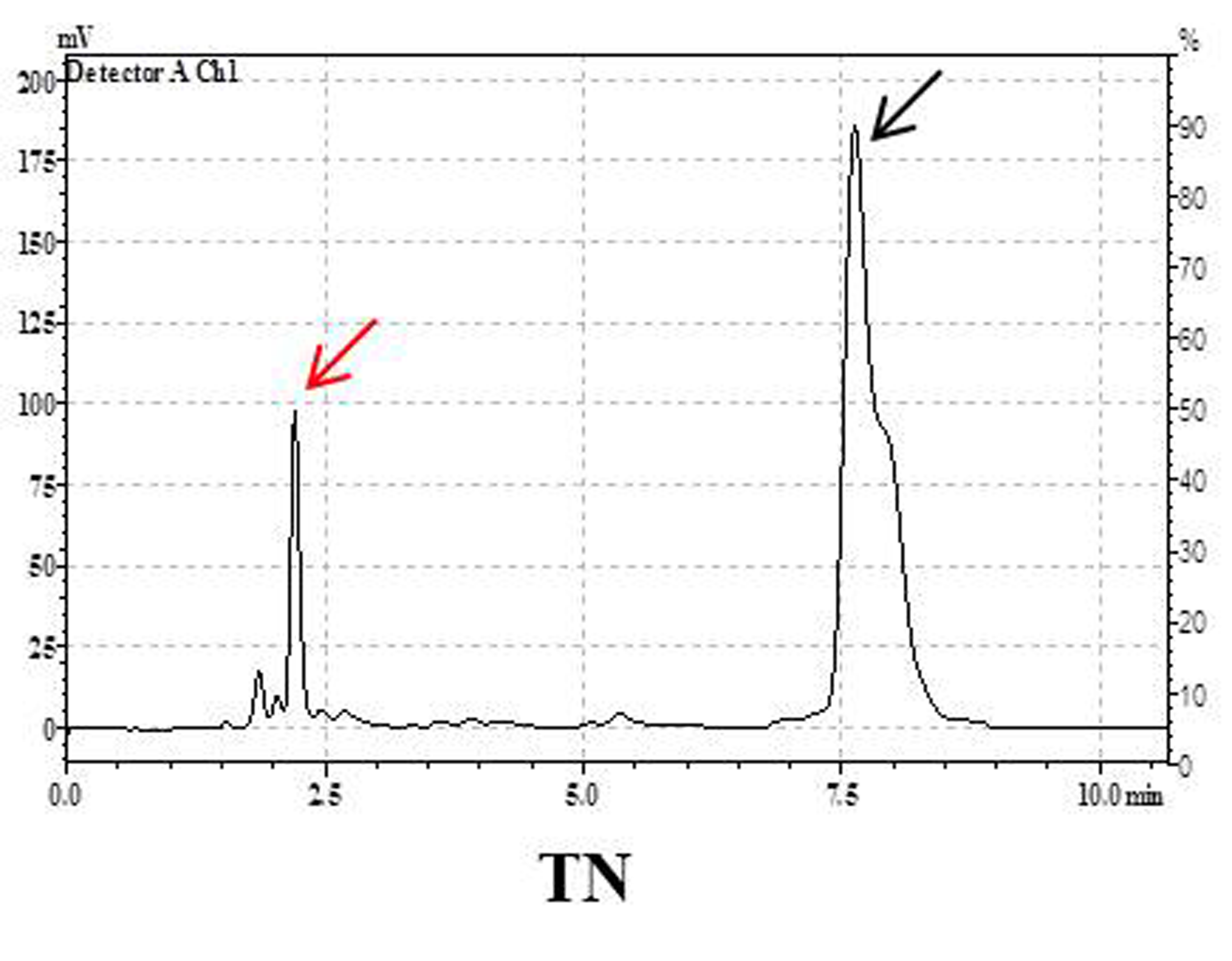


Fig. S2. HPLC chromatograph of *Dunaliella* cells were grown under the total nutrients (TN). Red arrow represents the lutein and black arrow represents the β-carotene.


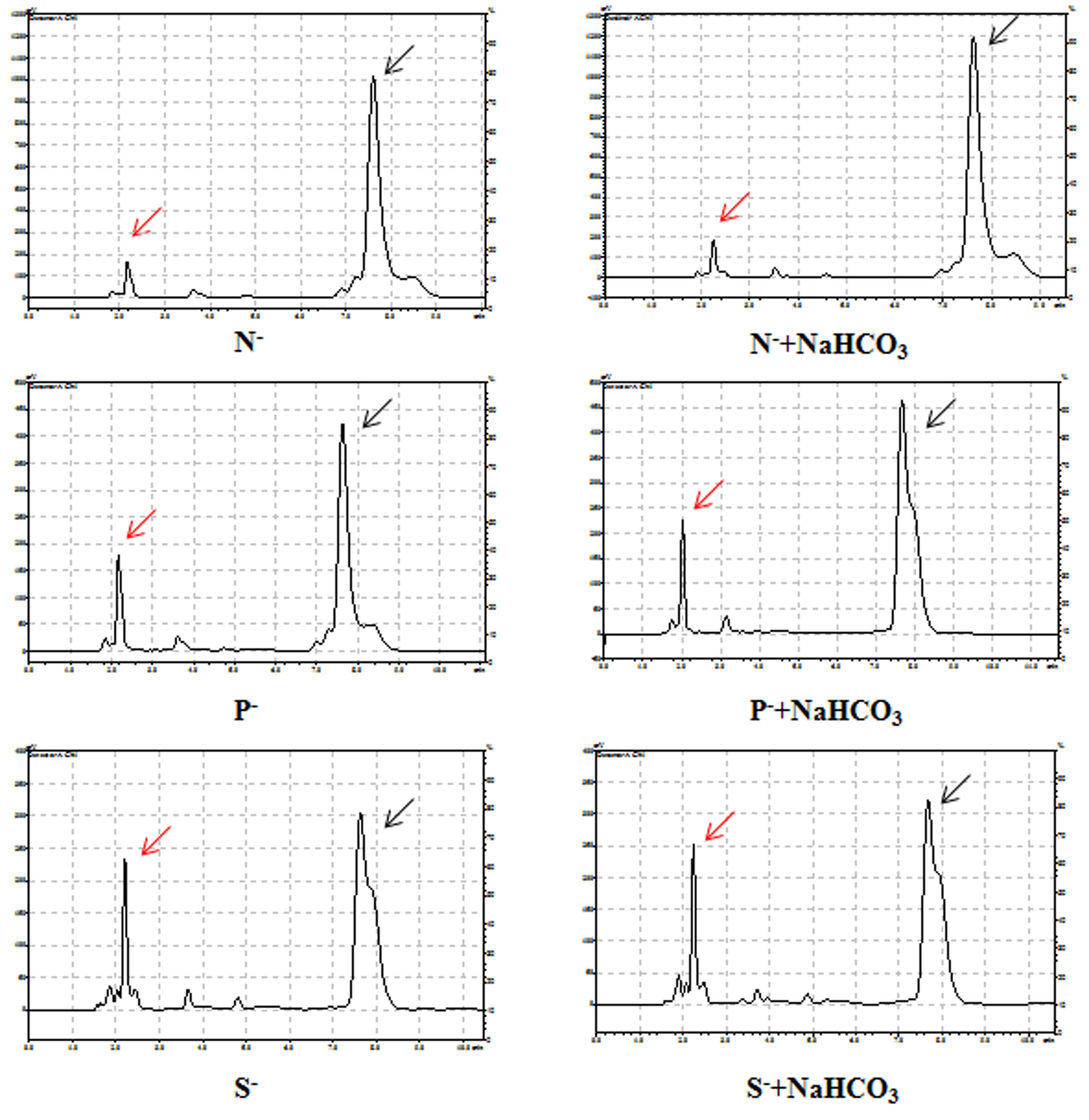
Fig. S3. HPLC chromatograph of *Dunaliella* cells were grown under the nutrient deficit conditions with or without sodium bicarbonate. Red arrow represents the lutein and black arrow represents the β-carotene. Nitrate deficit (N^-^); Nitrate deficit with bicarbonate (N^-^+NaHCO_3_); Phosphate deficit (P^-^); Phosphate deficit with bicarbonate (P^-^+ NaHCO_3_); Sulphate deficit (S^-^); Sulphate deficit with bicarbonate (S^-^+ NaHCO_3_).

| **FAMEs (%)** | **TN** | **N^-^** | **N^-^+NaHCO_3_** | **P^-^** | **P^-^+NaHCO_3_** | **S^-^** | **S^-^+NaHCO_3_** |
| --- | --- | --- | --- | --- | --- | --- | --- |
| Methyl12-methyl-tridecanoate | 0.67 ± 0.04^b^ | 1.12 ± 0.14^a^ | 1.25 ± 0.02^a^ | 0.37 ± 0.07^b^ | 0.57 ± 0.05^b^ | 0.47 ± 0.03^b^ | 0.45 ± 0.03^b^ |
| Tetradecanoic acid, 10,13-diethyl, methyl ester | 15.3 ± 0.11^e^ | 22.4 ± 0.01^b^ | 28.9 ± 0.21^a^ | 20.9 ± 0.12^c^ | 21.4 ± 0.06^c^ | 16.8 ± 0.10^d^ | 17 ± 0.09^d^ |
| Methyl10-trans, 12-cis-octadecadienoate | 0.58 ± 0.02^c^ | 1.62 ± 0.02^a^ | 1.65 ± 0.01^a^ | 0.87 ± 0.04^b^ | 0.89 ± 0.01^b^ | 0.62 ± 0.02^c^ | 0.56 ± 0.02^c^ |
| Methyl 7,11,14-eicosatrienoate | 2.81 ± 0.14^c^ | 4.78 ± 0.23^b^ | 5.96 ± 0.19^a^ | 2.97 ± 0.08^c^ | 3.25 ± 0.13^c^ | 2.79 ± 0.11^c^ | 2.81 ± 0.13^c^ |
| methyl 16-methyl-heptadecanoate | 20.3 ± 1.42^b^ | 24.3 ± 0.92^ab^ | 30.9 ± 1.78^a^ | 20 ± 0.84^b^ | 21 ± 0.97^b^ | 20.0 ± 2.51^b^ | 18.9 ± 1.67^b^ |
| Heptacosanoic acid, 25-methyl, methyl ester | 0.27 ± 0.01^d^ | 0.91 ± 0.02^ab^ | 1.02 ± 0.04^a^ | 0.27 ± 0.05^d^ | 0.69 ± 0.01^bc^ | 0.34 ± 0.15^d^ | 0.39 ± 0.07^cd^ |

Table. S1. Effect of bicarbonate on relative percentage of Fatty Acid Methyl Esters (FAMEs) extracted from lipids of *D. salina* under nutrient deficit conditions.

All values are expressed as Mean±SD (n=3). Values with different letters represent significantly differ at p<0.05 between the groups. Total Nutrient (TN); Nitrate deficit (N^-^); Nitrate deficit with bicarbonate (N^-^+NaHCO_3_); Phosphate deficit (P^-^); Phosphate deficit with bicarbonate (P^-^+ NaHCO_3_); Sulphate deficit (S^-^); Sulphate deficit with bicarbonate (S^-^+ NaHCO_3_).
